# Utilizing a Pathomics Biomarker to Predict the Effectiveness of Bevacizumab in Ovarian Cancer Treatment

**DOI:** 10.3390/bioengineering11070678

**Published:** 2024-07-03

**Authors:** Patrik Gilley, Ke Zhang, Neman Abdoli, Youkabed Sadri, Laura Adhikari, Kar-Ming Fung, Yuchen Qiu

**Affiliations:** 1 School of Electrical and Computer Engineering, University of Oklahoma, Norman, OK 73019, USAneman.abdoli@ou.edu (N.A.);; 2 Stephenson School of Biomedical Engineering, University of Oklahoma, Norman, OK 73019, USA; 3 Department of Pathology, University of Oklahoma Health Sciences Center, Oklahoma City, OK 73104, USAkarming-fung@ouhsc.edu (K.-M.F.)

**Keywords:** pathomics, digital histopathology, whole slide images

## Abstract

The purpose of this investigation is to develop and initially assess a quantitative image analysis scheme that utilizes histopathological images to predict the treatment effectiveness of bevacizumab therapy in ovarian cancer patients. As a widely accessible diagnostic tool, histopathological slides contain copious information regarding underlying tumor progression that is associated with tumor prognosis. However, this information cannot be readily identified by conventional visual examination. This study utilizes novel pathomics technology to quantify this meaningful information for treatment effectiveness prediction. Accordingly, a total of 9828 features were extracted from segmented tumor tissue, cell nuclei, and cell cytoplasm, which were categorized into geometric, intensity, texture, and subcellular structure features. Next, the best performing features were selected as the input for SVM (support vector machine)-based prediction models. These models were evaluated on an open dataset containing a total of 78 patients and 288 whole slides images. The results indicated that the sufficiently optimized, best-performing model yielded an area under the receiver operating characteristic (ROC) curve of 0.8312. When examining the best model’s confusion matrix, 37 and 25 cases were correctly predicted as responders and non-responders, respectively, achieving an overall accuracy of 0.7848. This investigation initially validated the feasibility of utilizing pathomics techniques to predict tumor responses to chemotherapy at an early stage.

## 1. Introduction 

Ovarian cancer currently stands as the leading cause of gynecologic cancer fatalities in women in the United States. The American Cancer Society estimates that 19,710 women were diagnosed with ovarian cancer in 2023, with an estimated 13,270 women perishing from the disease in the same year [1,2]. Approximately 90% of these ovarian cancer cases are typically diagnosed as epithelial ovarian cancer (EOC), the majority of which are high-grade serous tumors with a poor prognosis [2,3]. Due to a lack of effective screening methods [2,4] and asymptomatic presentation of early-stage ovarian cancers, many patients are diagnosed in the advanced stages of the disease [3,5,6]. The primary method for treating advanced stage ovarian cancer relies on cytoreductive debulking surgery to remove tumors, followed by chemotherapy treatments to decimate any surviving cancer cells [7,8]. According to Narod, one of the most important factors in determining whether a patient will survive ovarian cancer is how completely the chemotherapy treatments eradicate the surviving cancer cells [7]. Some cancer cells are resistant to chemotherapy agents, and if they survive treatment, then the recurring cancer will inherit this resistance. Therefore, choosing an appropriate chemotherapy agent is paramount in creating an effective treatment plan for patients.

Targeted agents are used to limit the growth and spread of tumor cells before debulking surgery and primary chemotherapy are administered to a patient [8]. The FDA recently approved bevacizumab for treating advanced ovarian cancer in combination with primary chemotherapy [9]. Bevacizumab is a monoclonal antibody that targets vascular endothelial growth factor (VEGF) and inhibits tumor angiogenesis [8,9]. It is effective in treating ovarian cancers that overproduce VEGF, such as EOC and peritoneal serous papillary carcinoma (PSPC) [10,11]. However, bevacizumab has the potential for serious side effects, such as delayed wound healing, hypertension, and bowel perforation [8]. Therefore, predicting the therapeutic effectiveness of bevacizumab is critical to conducting a benefit–risk assessment of administering it to each cancer patient. Several predictive markers have been explored for this purpose, including IL-6 levels [12], IL-8 gene polymorphism [13], adiposity [14], and VEGF-A protein expression [15,16]. However, most of these markers focus on the genetic and biological characteristics of ovarian cancer tumors. This neglects the tumor’s cellular and structural characteristics, which can vary greatly due to the heterogeneous presentation of ovarian cancers [17].

Pathomics is an emerging image analysis technology that focuses on extracting quantitative features that can characterize the diverse tissue types captured in digital pathology images [18,19,20]. Histopathological examination of tumor specimens is considered the gold standard of tumor diagnosis, making pathomics an effective tool for deriving valuable prognostic information about individual tumors from digitized tumor sample images [19]. Several studies have been conducted into applying pathomics to predict bevacizumab effectiveness in treating ovarian cancer [21,22,23]. However, most of these studies focused exclusively on the use of deep learning methodologies in conjunction with features derived solely from tumor tissues. In this investigation, we aimed to develop and evaluate the utility of a machine learning-based pathomics model that utilizes features derived from all available tissues within ovarian tumor samples in predicting the effectiveness of bevacizumab treatments. We developed an automated image analysis pipeline to segment ovarian tumor sample histopathology images and extracted quantitative image features from them. The extracted feature set was assessed via mutual information to identify the most valuable features; then, predictive models were trained to assess the performance of the identified features in predicting patient outcomes on an established ovarian cancer histopathology database.

## 2. Materials and Methods

### 2.1. Image Dataset

Our study utilized a digital histopathology whole slide image (WSI) dataset acquired from the Cancer Imaging Archive (TCIA) repository [6]. The dataset contained 288 de-identified WSI drawn from 78 patients who were treated using bevacizumab: 70 patients were diagnosed with epithelial ovarian cancer (EOC), and 8 patients were diagnosed with peritoneal serous papillary carcinoma (PSPC). The dataset was comprised of 162 WSI drawn from 44 patients where the treatment was effective and 126 WSI images spread across 35 patients where it was invalid. The WSI images were generated from tissue blocks of post-treatment specimens. All ovarian tumor specimens were surgically extracted and stained with hematoxylin and eosin (H&E) dyes using an automated slide stainer (ST5010 Autostainer XL, Leica, Wetzlar, Germany). A digital slide scanner (Leica AT Turbo, Leica, Wetzlar, Germany) equipped with a 20× objective lens was used to acquire the WSI images [6]. Many patients had multiple WSI associated with their records, so a single representative image was selected by expert reviewers for each case to reduce the computational time. The selection process emphasized histopathology images that minimized image artifacts and defocus while maximizing the quality of the H&E staining and tumor cell contrast. All available patient and clinical variables for the dataset were acquired from the available image metadata in the TCIA repository. 

### 2.2. Histopathology Image Feature Extraction

**Figure 1:**
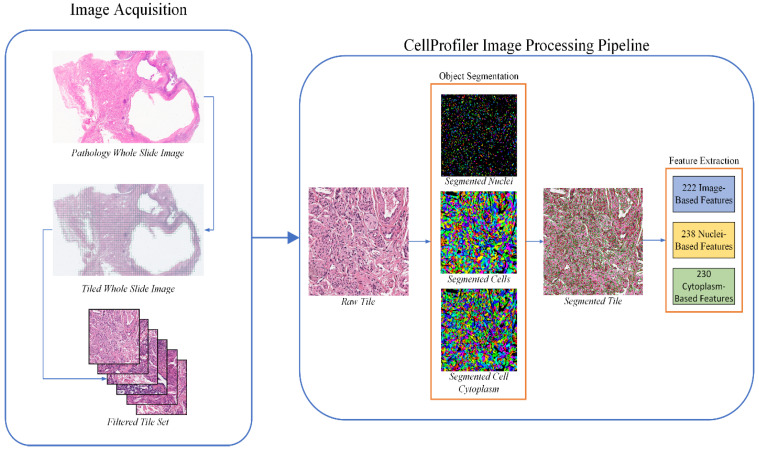
Workflow of the proposed treatment effectiveness prediction strategy. The left side shows the acquisition and pre-processing of whole slide images (WSIs). The original WSI is sliced into non-overlapping tiles, which are filtered into a useful image set based on tile quality and tissue content. The right side shows the CellProfiler image segmentation and feature extraction process. The pipeline processes each individual tile, identifying tumor nuclei, cells, and cytoplasm within the image tile. The images within the orange “Object Segmentation” section are binary object masks that are randomly colored to distinguish neighboring objects. The “Segmented Tile” image shows the colored outlines of all segmented objects, with nuclei outlines in red and cell outlines in green. Cytoplasm is treated as the area between a green outline and the red outline inside of it. Once the segmentation process is completed, features are extracted from the object sets and the original image.

In order to extract useful quantitative image features from our representative WSI case images, we constructed an automated image processing pipeline using CellProfiler to segment and extract features from sample tissue, cell nuclei, and cell cytoplasm. Figure 1 shows the general workflow for processing each representative WSI using our method. The image processing workflow is separated into two major tasks: pre-processing the WSI and segmenting and extracting features from the processed images using our CellProfiler pipeline. Before executing our segmentation and feature extraction process, each WSI was split into non-overlapping 1000 × 1000 image tiles. Our study only considered image tiles which contents were at least 50% tissue. All tissue contained within each WSI was considered; no distinction was made between healthy tissue and tumor tissue. The tiling process is shown in Figure 1 as a grid overlaid on a sample WSI, where each grid square shows an image tile that satisfies the tissue threshold. A manual review was conducted of the final image tile sets using criteria similar to our case representative image selection process to discard any image tiles containing significant tissue artifacts.

Once the usable tile sets were constructed for our representative WSI, they were analyzed by our CellProfiler pipeline. Figure 1 illustrates the overall process of the CellProfiler pipeline, which adapted an approach used by [24] to segment and predict the prognosis of non-small cell lung cancer. The first step of the pipeline split each image tile by stain using spectral deconvolution, creating greyscale images of the hematoxylin and eosin stains used in each WSI sample. All features related to texture and intensity were computed using these stain images. An adaptive cross-entropy threshold was used to identify and remove any tissue folds present in the image tiles from further analysis in the pipeline based on their disproportionally heavy staining. Next, an Otsu threshold was used to segment the cell nuclei. These segmented nuclei were then used as seeds for a propagation algorithm using a minimum cross-entropy threshold to identify entire cells. Cytoplasm was defined as cell regions that were not contained within the cell’s nuclei; thus, cytoplasm was segmented by subtracting nuclei regions from their matching whole cell regions. This process is shown in Figure 1 in the images within the orange rectangle. These images contain binary masks of identified objects, randomly colored to differentiate separate objects. The top image shows the nuclei segmented from a sample image tile, and the middle image shows the cells identified by CellProfiler from the nuclei seeds. The bottom image shows cytoplasm objects segmented by subtracting the top nuclei image from the middle cell image.

The pipeline was designed to extract a wide array of feature types in order to better identify an effective ovarian cancer diagnostic feature set. The quantitative features extracted by the pipeline can be broken down into two broad categories: image-based and object-based. The image-based features measure each image tile as a whole. These features focus on the texture, intensity, granularity, image correlation between stain images, and the overall image quality. Object-based features measure the intensity, radial intensity distribution, texture, size, and shape of segmented cell nuclei and cytoplasm, as well as the spatial relationship between neighboring nuclei. In total, the pipeline extracted a total of 690 unique feature measurements from each image tile. The breakdown of these features is shown in Figure 1. Image-, nuclei- and cytoplasm-based features account for 222, 238 and 230 unique features, respectively. Note that, while image-based features were only measured once per image tile, each object-based feature was measured for every segmented object belonging to a specific class. For example, the 238 unique nuclei features are measured for every segmented nucleus, producing a unique feature vector for each one. The features were then aggregated across all tiles belonging to each representative WSI by their mean, median, standard deviation, and decile values. This process created case-level feature vectors by pooling all the data associated with each case/representative image. For example, deriving nuclei-based features from a representative WSI would calculate the statistical measures by using data from every cell nucleus segmented in the image tiles, regardless of which tile any specific cell nucleus was segmented from. Likewise, case-level image-based features are derived by pooling the image measurements from all the tiles into a single population, then using that data to calculate the statistical measures. After feature aggregation, each case in the OBR dataset had 9828 features associated with it. We discarded duplicate and low variance features, reducing our dataset to 7941 features for each case.

### 2.3. Model Training

In order to improve the performance of our trained models, we evaluated all 7941 features using mutual information (MI). We calculated the MI scores between our feature vectors and class labels, then established MI selection thresholds to determine which features to keep using the methods in [25]. All features with a MI score underneath the threshold were discarded. It was also important to balance the number of selected features against the number of cases in our dataset to ensure optimal performance from our models. Therefore, we employed an MI threshold of 0.29, which corresponded to selecting the top 100 features of our dataset. We trained our models using these 100 features to classify patients by whether their chemotherapy treatments were effective or invalid. Two support vector machine (SVM) models were trained to predict the treatment outcomes of the OBR dataset patients. An SVM is a robust supervised learning technique that, when applied to classification tasks, generates a hyperplane that optimally separates the classes in a dataset. This process optimizes the hyperplane by maximizing the margins or distance between the hyperplane and the nearest data vectors [26,27,28]. An SVM model can be expressed mathematically as an attempt to optimize Equation (1):

(1)  min ω,C,ξ ⁡ 12ωTω+C∑i=1lξi  subject to yiωTϕxi+b≥1−ξi, ξi ≥0, i=1,…,l

where ω is the normal vector, C is the coefficient regularization parameter, ξ is the constraint slack variable, and $x_{i}$ and $y_{i}$ represent the training vectors and vector labels, respectively. An SVM kernel function is used to transform the feature space in order to better separate nonlinear data. Our study utilized the linear and Gaussian radial basis function (RBF) kernels, which are shown below as Equations (2) and (3), respectively.

(2) $$\;K\left(x_{i},x_{j}\right)=x_{i}\cdot x_{j}$$

(3) $$K\left(x_{i},x_{j}\right)=\exp\left(-{\gamma \left\|x_{i}-x_{j}\right\|}^{2}\right)$$

In Equations (2) and (3), $x_{i}$ and $x_{j}$ represent the feature vectors, and γ controls the sensitivity of the RBF kernel.

The SVM models were trained and evaluated using a nested 5-fold cross-validation approach. Five-fold cross-validation is a robust method of training predictive models to assess how well they generalize to unseen data [29]. It randomly partitions a dataset into 5 groups of samples, referred to as folds. A single fold is held out as the testing data, and the remaining folds are used as the training data. This process is repeated until all folds have been used as testing data. Nested cross-validation is a multi-layered approach that optimizes the model hyperparameters during the model training process [30]. This approach was employed to tune the C coefficient regularization hyperparameter of our SVM models using a grid search approach. Both models were evaluated using receiving operating characteristic (ROC) curves, area under the ROC curve (AUC) values, and overall model prediction accuracies.

## 3. Results

**Figure 2:**
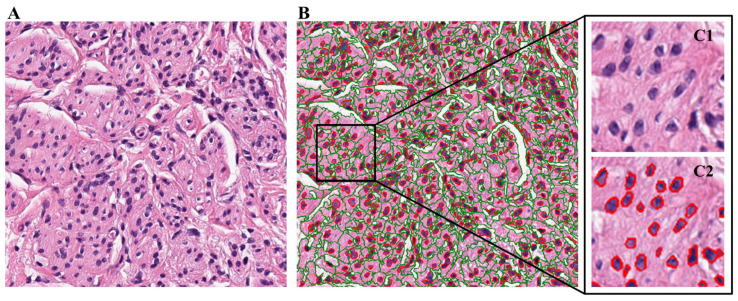
Example results of the tissue tile segmentation process. (**A**) Original tissue tile image. (**B**) Tissue tile image after segmentation by CellProfiler. Segmented nuclei objects are outlined in red, and segmented cell objects are outlined in green. Cytoplasm is defined as the region between a green outline and the red outline inside of it. (**C1**,**C2**) Enlarged sample region of the segmented tile image. (**C1**) The original, unsegmented tissue. (**C2**) The cell segmentation results.

Example results of the CellProfiler pipeline’s nuclei and cytoplasm segmentation process are shown below in Figure 2. Figure 2A shows a raw image tile extracted from an invalid treatment case WSI. The segmentation results of the CellProfiler pipeline are depicted in Figure 2B. Each object is shown using different outlines, where red outlines represent segmented nuclei, and green outlines represent segmented cells. Cytoplasm was interpreted as the area between a green outline and the red outline inside of it. Figure 2(C1,C2) show an enlarged area of Figure 2B and demonstrate each step of the object segmentation process. Figure 2(C1) shows the original, unsegmented version of the image, and Figure 2(C2) shows the image after the cell nuclei were segmented.

**Figure 3:**
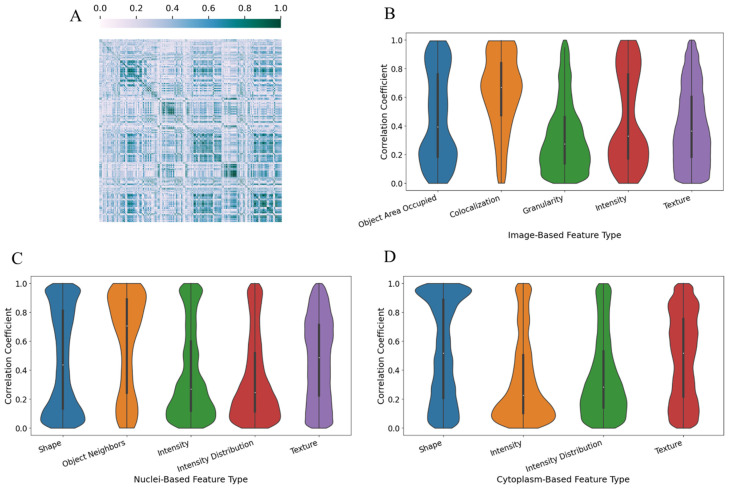
Correlation analysis of the features after removal of duplicate and near-zero variance features. (**A**) Heatmap of the 7941 analyzed feature correlation coefficients. (**B**–**D**) Violin plots of the distribution of the correlation coefficients in the selected feature types. The feature types are grouped by their feature classes: (**B**) image, (**C**) nuclei, and (**D**) cytoplasm.

Figure 3A shows a heatmap of the 7941 features used to train our models. Each cell in the heatmap represents the Pearson correlation coefficient (PCC) values between two features, with white representing low PCC values and green representing high PCC values. The heatmap is divided by feature classes: from left to right, they are image-based, nuclei-based, and cytoplasm-based features. The interdependency of the features in the dataset is influenced by both the type and class of each individual feature. The feature types with the strongest correlations within our dataset were the Zernike shape features of the segmented objects and the texture features of the image and segmented objects. These features showed a strong interdependency within the nuclei and cytoplasm feature classes; however, they showed weak correlations between the two feature classes. The texture features were the other feature type to show strong correlations within our dataset. The texture features for the cytoplasm, image, and nuclei feature classes were highly interdependent within each feature class. However, unlike the Zernike shape features, the texture features showed significant correlations between feature classes. Figure 3B–D illustrate the distribution of correlation coefficients within each feature class. These figures only show select feature types for each class; they are not a comprehensive list of all feature types for each feature class. Figure 3B shows several trends within the image-based features. Most feature types skew towards one end of their correlation distributions, with colocalization features showing strong associations while texture and granularity features show weak associations. However, the intensity and image area occupied by object features are distributed towards both ends of their distributions, showing strong and weak associations in equal measure. These relationships also occur in the object-based features shown in Figure 3C,D. The intensity and radial intensity distribution features in both figures show weaker associations. The texture features show no overall pattern within either object-based feature class. Shape and object neighborhood features exhibit both strong and weak correlations, though cytoplasm-based shape features trend towards stronger correlations than nuclei-based shape features.

**Figure 4:**
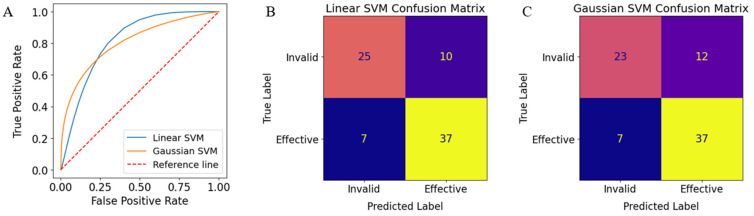
SVM prediction performance. (**A**) ROC curves for the linear and Gaussian SVM models. (**B**,**C**) Sample confusion matrices for the linear and Gaussian SVM predictions, respectively.

Generally, the top 100 features of our dataset included Haralick texture features of the nuclei (InfoMeas1 and InfoMeas2) and cytoplasm (InfoMeas2 and DifferenceEntropy), radial distribution of the intensities in the nuclei and cytoplasm, and Zernike and object size measurements of the nuclei and cytoplasm. Table 1 shows the area under the ROC curve (AUC) values and accuracies of the SVM models, along with their precision, recall, and F-scores. Figure 4 shows the average performance for both linear and Gaussian SVM models. Figure 4A shows the receiving operator characteristic (ROC) curves for both models. The linear and Gaussian SVM models achieved AUC values of 0.8312 ± 0.0462 and 0.8253 ± 0.0470, respectively. Figure 4B shows the confusion matrix for the linear SVM model. Out of 47 cases where the linear SVM predicted that the treatment was “Effective”, 37 cases were confirmed as being responsive to treatment based on their 6-month progression-free survival (PFS) data. Similarly, out of 32 cases where the treatment was predicted as “Invalid”, 25 cases were confirmed to be non-responsive to treatment. These results yielded a positive predictive value (PPV) of 78.72% and a negative predictive value (NPV) of 78.13% for the linear SVM model, corresponding to a total accuracy of 78.48%. Figure 4C shows the confusion matrix for the Gaussian SVM model. Evaluating Figure 4C with the same methods as Figure 4B shows that the Gaussian SVM achieved an overall accuracy of 75.95%. The linear and Gaussian SVM models produced identical recall values; however, the linear SVM model outperformed the Gaussian SVM model in every other performance metric.

## 4. Discussion

In this study, we developed and initially validated the ability of pathomics technology to predict the outcome of administering bevacizumab treatments to ovarian cancer patients. As compared to previous research in this area, the major unique characteristic of this study is the utilization of histopathological information to predict treatment effectiveness. Most of the previous research into predicting the efficacy of various cancer therapies has relied on clinical, molecular, and imaging (CT and MRI)-based biomarkers [31]. Especially on the CT/MRI images, the treatment effectiveness can be directly evaluated by visually evaluating the tumor changes before and after administered treatment therapies [32]. In clinical practice, the histological evaluation of tumor tissues is one of the most basic routine examinations for cancer patients, which is widely accessible in most hospitals. However, the visual interpretation of these histopathology slides can only determine the tumor histology or tumor type, and their association with therapy responses are not consistent, as both positive and negative results have been reported [33,34]. This visual reading also suffers from low inter-pathologist agreement. Meanwhile, these collected histopathology slides also contain more clinically meaningful information for determining a tumor prognosis, which can be extracted and organized using novel pathomics approaches. This information is identified by characterizing the tumor cell nuclei/cytoplasm in density, heterogeneity, spiculation, surrounding geometry, etc. Accordingly, in this study, these tumor characteristics were quantified by a large amount of cell/cytoplasm features, based on which an SVM model was developed to evaluate treatment effectiveness. The best model trained in this study achieved an accuracy of 78.48% when predicting bevacizumab treatment outcomes on a database of 78 patient WSIs, which initially validated our hypothesis. 

To the best of our knowledge, this is the first time that a very large amount of pathomics features was utilized to predict tumor responses to chemotherapy, even though the feature extraction pipeline used in this study was adapted from a study for diagnostic and prognostic evaluations of non-small cell lung cancer [24]. All these features indicate highly complicated correlation trends, which implies the complexity of the cell/cytoplasm morphology on the histology slide. Specifically, among the selected best-performing features, the texture and Zernike features of both the nuclei and cytoplasm, as well as the radial intensity distribution of the cytoplasm, were commonly selected. This suggests that pathomics features that extract information about subcellular structures that are useful in predicting survival rates are also relevant in evaluating the efficacy of specific treatment therapies, validating the feature analysis conducted in [24] for our models. Interestingly, the features selected in this study were remarkably similar to the features selected for both machine learning model applications evaluated in [24], implying these feature types are highly versatile and can generalize effectively to a broad range of tasks.

Although this study achieved encouraging results, there are several limitations to it that need to be acknowledged. First, the OBR database was collected from 78 patients treated at a single institution. Our models should be trained on data collected from multiple medical centers to verify their robustness and generalizability. Second, our models were only developed with conventional SVM models. Other machine learning technologies—in particular, deep learning [21,22]—were not explored in this study. Deep learning models and features have the potential to better exploit the complex feature space of pathomics-derived WSI features. Third, the thin 2D tissue slices contained in the OBR database WSIs do not provide much structural information, so augmenting our feature set with information from CT or MRI images has the potential to further improve the performance of our models. Despite the limitations of our study, our results show that pathomics and digital histopathology images will play a valuable role in precision treatments in oncology by providing valuable tools in tailoring treatments to specific presentations of ovarian cancer.

## 5. Conclusions

Histopathological images contain copious and clinically meaningful information to predict the effectiveness of bevacizumab therapy for ovarian cancer patients. This meaningful information can be effectively extracted and identified via the use of pathomics methods, which has the potential to be applied in future clinical practice.

## Figures and Tables

**Table 1:** Quantitative performance metrics of the SVM models.

Model Type	AUC	Accuracy	Precision	Recall	F-Score
Linear	0.8312	0.7848	0.7872	0.8409	0.8132
Gaussian	0.8253	0.7595	0.7551	0.8409	0.7957

## Data Availability

The research data generated in this study is not publicly available.
